# A Smartphone App for Self-Monitoring of Rheumatoid Arthritis Disease Activity to Assist Patient-Initiated Care: Protocol for a Randomized Controlled Trial

**DOI:** 10.2196/15105

**Published:** 2020-02-19

**Authors:** Bart F Seppen, Merel J L'ami, Sharon Duarte dos Santos Rico, Marieke M ter Wee, Franktien Turkstra, Leo D Roorda, Fabio S Catarinella, Dirkjan van Schaardenburg, Michael T Nurmohamed, Maarten Boers, Wouter H Bos

**Affiliations:** 1 Amsterdam Rheumatology and Immunology Center Reade Amsterdam Netherlands; 2 Department of Rheumatology VU Medical Center Amsterdam UMC Amsterdam Netherlands; 3 Department of Epidemiology & Biostatistics Amsterdam Public Health, Vrije Universiteit Amsterdam Amsterdam UMC Amsterdam Netherlands; 4 Department of Rehabilitation Reade Amsterdam Netherlands; 5 Brightfish BV Amsterdam Netherlands; 6 Department of Rheumatology Amsterdam Medical Center Amsterdam UMC Amsterdam Netherlands

**Keywords:** smartphone app, telemonitoring, rheumatoid arthritis

## Abstract

**Background:**

Telemedicine based on self-measurement of disease activity could be one of the key components to create the health care system of the future. Previous publications in various medical fields have shown that it is possible to safely telemonitor patients while reducing the number of outpatient clinic visits. For this purpose, we developed a mobile phone app for patients with rheumatoid arthritis (RA), which allows them to self-monitor their disease.

**Objective:**

The objective of this study is to assess the safety and efficacy of self-initiated care assisted by a smartphone app in patients with RA.

**Methods:**

This is a randomized controlled trial that will be performed for 1 year. A total of 176 patients with RA will be randomized to either self-initiated care with only one scheduled follow-up consultation assisted by our app or usual care. The coprimary outcome measures are the number of outpatient clinic consultations with a rheumatologist taking place during the trial period and the mean disease activity score as measured by the disease activity score 28 (DAS28) at 12 months. The secondary outcomes are patient satisfaction, adherence, patient empowerment, and cost evaluation of health care assisted by the app.

**Results:**

Recruitment started in May 2019, and up to 18 months will be required for completion of recruitment. Thus far, 78 patients have been randomized, and thus far, experiences with the app have been positive. The study results are expected to be published by the end of 2021.

**Conclusions:**

The completion of this study will provide important data regarding the following: (1) safety of self-initiated care supported by a smartphone app in terms of DAS28 and (2) efficacy of lowering health care usage with this new strategy of providing health care.

**Trial Registration:**

Netherlands Trial Register NL7715; https://www.trialregister.nl/trial/7715

**International Registered Report Identifier (IRRID):**

DERR1-10.2196/15105

## Introduction

### Background

Rising health care costs, increasing elderly population size, and shortage of medical personnel have forced us to think about alternative ways to organize our health care system. The use of information technology tools (eHealth) may lead to better outcomes while reducing costs [1]. One suggested use for eHealth is asynchronous telemonitoring. In this form of telemonitoring, patients are monitored without face-to-face or real-time contact with a physician. Various studies with asynchronous telemonitoring have been performed in patients with inflammatory bowel disease (IBD) [2], asthmatic diseases [3,4], and diabetes mellitus [5], with positive results. However, for patients with rheumatoid arthritis (RA), clinical evidence for the use and safety of asynchronous telemonitoring is lacking [6].

Currently, patients are monitored in outpatient clinics according to the European League Against Rheumatism (EULAR) treat-to-target guidelines for RA. The guidelines state that measures of disease activity must be obtained and documented regularly, as frequently as monthly for patients with high/moderate disease activity or less frequently, such as every 6 months, for patients with sustained low disease activity or those in remission [7]. On one hand, the value of most consultations is low, as 75% of patients in routine clinical follow-up have low disease activity or show remission [8]. On the other hand, individuals with RA may experience occasional increases in inflammation between routine clinical visits, which are associated with worsening symptoms, and these are referred to as flares [9]. Moreover, patients with RA characterize flares as unpredictable intense episodes that make them feel helpless [10]. Frequent self-monitoring of disease activity combined with self-initiated care could lead to the early identification of flares and treatment intensification while reducing appointment frequency for stable patients. Furthermore, with implementation of such measures, patients with RA may benefit from reduced travel time, less work leave, and possibly reduced health care costs.

There are already many smartphone apps available for self-monitoring of RA. However, high-quality apps are lacking [11]. For instance, integration with electronic medical record (EMR) systems is practically nonexistent. Therefore, we developed a new app. The developed app requests patients to fill in a Routine Assessment of Patient Index Data 3 (RAPID3) questionnaire weekly for self-measurement of RA disease activity [12,13]. If disease activity assessment indicates flares, patients are instructed to contact the outpatient clinic through the app. We expect the app to support self-initiated care and to help achieve better disease activity management between scheduled clinic visits.

### Objective

The objective of this paper is to report a protocol for a randomized controlled trial (RCT) that will test if we can safely (noninferiority in terms of Disease Activity Score [DAS] 28) reduce the number of outpatient clinic visits in patients with RA who self-monitor their disease. If the results confirm our hypothesis, we aim to implement our telemonitoring strategy in the Dutch health care system.

## Methods

### Overview

The study will take place at Reade, a secondary rheumatology clinic in Amsterdam, the Netherlands. Reade developed the smartphone app together with the software company Brightfish BV (Amsterdam, the Netherlands), which offers advice and assistance to ensure that important standards for cybersecurity, software design, and software maintenance are met [14]. The city of Amsterdam along with its surroundings is an ideal setting for this study, as network coverage in the Netherlands is excellent, reaching as high as 96.8%, overall, and 100% in Amsterdam [15]. Furthermore, over 87% of the Dutch adult population owns a smartphone, and the mobile download speed currently ranks sixth worldwide [16,17].

### Design, Test, and Redesign

**Figure 1:**
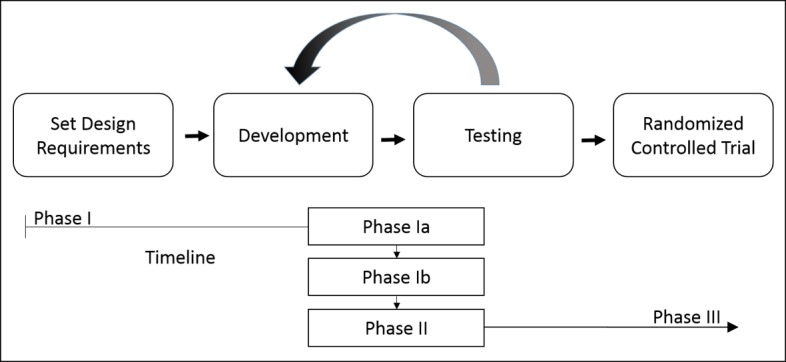
The design, test, and redesign approach in three distinct phases. In phase I, the app was developed and tested twice. A prepilot check (Ia) was performed to test usability. Subsequently, the app was tested in a mixed-methods pilot study (Ib). In phase II, further development was performed and the new app was re-evaluated with another mixed-methods pilot study (manuscript of pilot studies in preparation) [19].

Following the Medical Research Council guidance for developing and evaluating complex interventions, the development and evaluation of the app was carried out in three distinct phases, which will be described in detail elsewhere (manuscript in preparation) [18]. The process is presented in Figure 1. Only the protocol for an RCT (phase III) will be reported here. The manuscript of the development process and pilot studies is in preparation [19].

### Phase III: Protocol for a Randomized Controlled Trial

In the third phase of the development process, an RCT will be performed. In this 1-year study, the safety and efficacy of self-initiated care assisted by our app will be evaluated. The proposed work process has been thoroughly evaluated with physicians, researchers, and two patient partners. Multiple meetings have been held to adjust and improve the process, with involvement of patients and rheumatologists in each step. Ultimately, all stakeholders agreed on the strategy, and therefore, we anticipate successful implementation of this proposed renewed health care design, if it is clinically proven to be successful.

The trial has been registered at Trialregister.nl, a publicly available and freely searchable register for studies in the Netherlands. The study has been approved by the research ethics committee of the Amsterdam UMC. A total of 176 patients will be recruited at the outpatient clinic of Reade, a center for rehabilitation and rheumatology in Amsterdam, the Netherlands. As over 3000 patients with RA are currently receiving outpatient clinic care at Reade, adequate recruitment is expected to be feasible.

### MijnReuma Reade App (MyRheumatism App)

**Figure 2:**
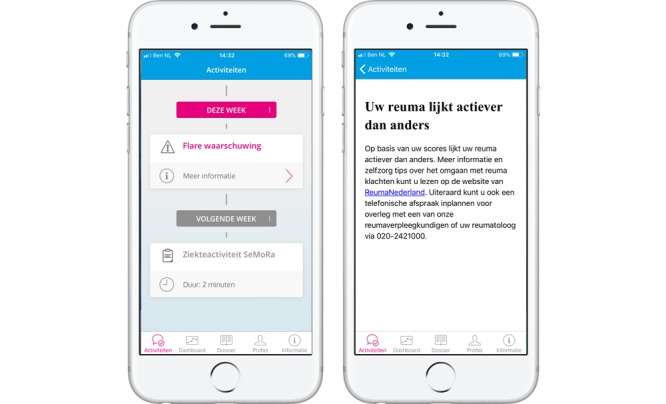
The MijnReuma Reade App. Five separate screenshots illustrate the simple and comprehensive design and interface of the app. The second screenshot from the left portrays one of the disease activity graphs.

**Figure 3:**
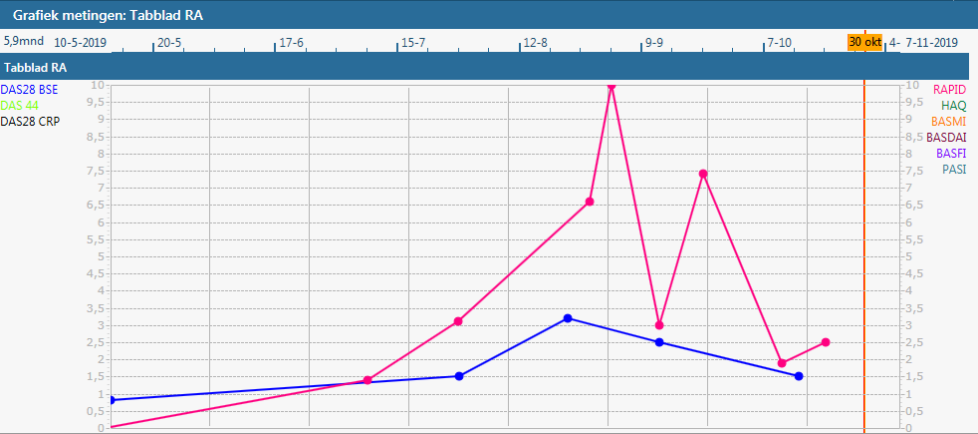
EMR dashboard. The EMR dashboard displays the results collected with the app. The data originate from the EMR itself, where they are stored in real-time after questionnaire completion in the app. The graph displays disease activity over time as measured by the RAPID3 (red) and DAS28 (blue). DAS28: Disease Activity Score 28; EMR: electronic medical record; RAPID3: Routine Assessment of Patient Index Data 3.

The built app aims to collect self-assessment questionnaire data every week (Table 1). The user is prompted by weekly reminders sent by the app to fill out the questionnaire. If the user does not fill out the questionnaire, another reminder is sent after 24 hours. If the questionnaire is still not complete, another reminder is sent 1 week after the initial reminder. The app presents outcomes over time in a graph and provides access to patient medical records and information regarding RA (Figure 2). When a user completes the questionnaire, the data from the app are transmitted securely (transport layer security) to secure servers on the hospital premises. The servers are connected to the Reade EMR system. Data handling is fully compliant with all relevant Dutch privacy and security laws, including ISO27001 and General Data Protection Regulations. In the EMR system, numerical scores and a graph of the self-assessment questionnaire data over time can be viewed by Reade health care professionals (Figure 3).

**Table 1:** Self-assessment questionnaire in the MijnReuma Reade App.

Domain	Measure	Number of questions
Function^a^	mHAQ^b^	10
Pain^a^	NRS^c^ (0-10)	1
Patient-Global^a^	NRS (0-10)	1
Fatigue	NRS (0-10)	1
Morning stiffness	Minutes	1
Social participation	Likert scale (0-3)	1
Sleep	Likert scale (0-3)	1
Anxiety	Likert scale (0-3)	1
Stress	Likert scale (0-3)	1
Flare question	Yes/no	1

^a^These items together form the Routine Assessment of Patient Index Data 3.

^b^mHAQ: modified health assessment questionnaire.

^c^NRS: numeric rating scale

### Algorithm

**Figure 4:**
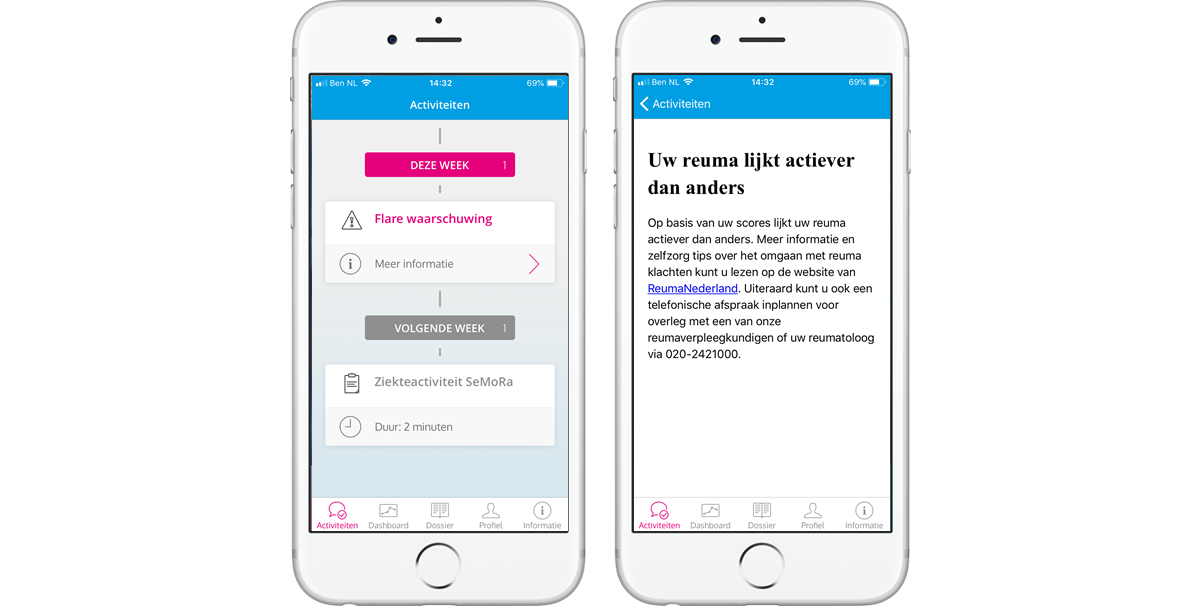
Flare algorithm. The app generates an alert with a link to self-management tips and advice to contact the outpatient clinic in case of an increased disease activity. Left: "Flare warning". Right: "Your disease appears to be more active".

The app sends an alert to the user when the algorithm recognizes a flare (Figure 4). The alert notifies the user of a possible flare, provides links to self-management tips, and advises the user to contact a rheumatology nurse if necessary. The alert is generated when the RAPID3 value of the user increases by more than 2 points from the previous value and the current RAPID3 value is >4 points. This threshold has been determined on the basis of the findings in previous studies, in which a flare according to the DAS28 corresponded with a RAPID3 increase between 1.5 and 2.3 points [9,20]. Furthermore, the cutoff of 4 points corresponds very well to the patient- and physician-defined flare cutoffs of 4.33 and 4.27, respectively [20]. Receiver operating characteristic curve analysis showed that a RAPID3 value >4.27 had 77.3% sensitivity and 77.6% speciﬁcity for patient-defined flare and that a cutoff of 4.33 had 67.6% sensitivity and 85.3% speciﬁcity for physician-defined ﬂare. The discussed alternatives included the RA flare questionnaire (RA-FQ) and FLARE-RA score [9,21]. As remission criteria are not available for both these assessments, three out of five RA-FQ domains are captured with the RAPID3, and RAPID3 values and FLARE-RA scores are highly correlated (*r*=0.77), it was ultimately decided to use the RAPID3-based algorithm [22]. An anchor question regarding the presence of an RA flare (yes/no) was included in the weekly self-assessment questionnaire (Table 1) to evaluate the appropriateness of the threshold in this study [9].

### Eligibility Criteria

The inclusion criteria are as follows: diagnosis of RA by a rheumatologist; disease duration of at least 2 years; low disease activity or remission (DAS28 <3.2) at the time of inclusion; use of a disease-modifying antirheumatic drug (DMARD); owning a mobile device with an Android or iOS operating system (implying mobile phone literacy); age of at least 18 years; and ability to read and speak Dutch.

The exclusion criteria are as follows: medication change involving initiation or discontinuation of a DMARD (conventional [methotrexate, cyclosporine, cyclophosphamide, gold injections, hydroxychloroquine, leflunomide, mycophenolate, sulfasalazine, and corticosteroids] or biological) in the last 6 months and participation in another interventional study.

### Study Design

Outpatient clinic patients will be informed about the study by their rheumatologist. If consent is given, they will be contacted by the trial physician (BS). They will be invited for a study visit to discuss the study, sign the informed consent form, and undergo screening for eligibility. The selected patients will be randomized to one of the following two parallel groups: control group and intervention group. Patients randomized to the control group will continue usual care, and outpatient clinic visits are planned as usual by their rheumatologist, on average 2-3 times a year. For patients randomized to the intervention group, only one outpatient clinic visit is planned at the end of the trial period (after 12 months). They will receive a username and password for the app to allow monitoring of their own symptoms. If necessary, they will be provided help to download the app from the appropriate app store. During the first study visit, they will be instructed on how to complete the weekly questionnaire through the app. Additional follow-up visits will be scheduled at the occurrence of flares as recognized by the app or at the request of individual patients. Furthermore, at 6 months of the intervention, the blood of the patients will be tested (erythrocyte sedimentation rate [ESR], hemoglobin level, leukocyte count, aspartate aminotransferase level, and alanine aminotransferase level) at the outpatient clinic. Patients will receive the results of the blood tests over the phone. If trial patients do not complete the weekly questionnaire for 4 weeks, they will be contacted to investigate the reason for nonadherence. Nonadherence will not lead to discontinuation of the trial for trial patients.

After 12 months, all patients will be seen by the trial physician for the second and final study visit. During the visit, a tender and swollen joint count will be performed by a blinded research nurse or a rheumatology resident. For patients in the intervention group, the number of flare reports will be collected and, if applicable, the reason for not contacting the outpatient clinic will be recorded.

**Figure 5:**
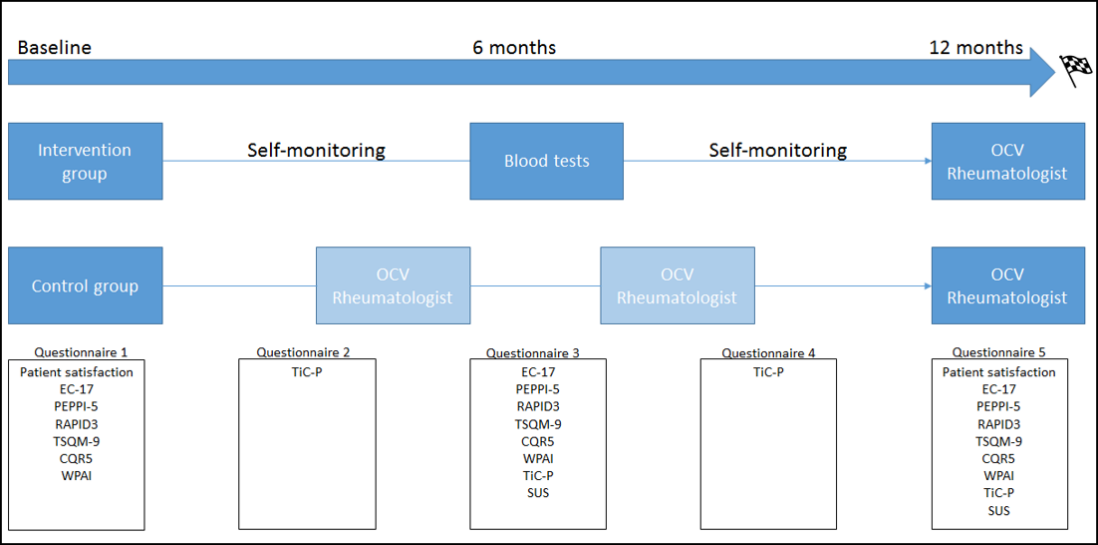
Study design and outcomes over time. The control group continues regular care, usually with two or three preplanned outpatient clinic visits (OCVs) (light blue box). The intervention group has only one preplanned OCV (blue box) and monitors symptoms using a smartphone app. All patients complete five questionnaires during the study to compare secondary outcomes. CQR5: Compliance Questionnaire for Rheumatology 5; EC-17: Effective Consumer Scale 17; PEPPI-5: 5-item Perceived Efficacy in Patient-Physician Interactions; RAPID3: Routine Assessment of Patient Index Data 3; SUS: System Usability Scale; TiC-P: Treatment Inventory of Costs in Psychiatric Patients; TSQM-9: Treatment Satisfaction Questionnaire for Medication 9; WPAI: Work Productivity and Activity Impairment.

During the trial, all patients will complete validated self-reported questionnaires at 0, 3, 6, 9, and 12 months, using a web-based system (Figure 5).

### Flare

All patients, irrespective of the algorithm results or randomization arm, will be allowed acute outpatient visits if needed. If patients seek health care during the study, they will be seen by a rheumatology nurse. Patients will be asked about possible flares, associated symptoms, and medication adherence. If the rheumatology nurse cannot manage the flares, the patient’s own rheumatologist or the on-call rheumatologist (when the patient’s own rheumatologist is absent) will be notified by the rheumatology nurse for further treatment.

### Power Analysis

To evaluate the safety of app-supported self-initiated care, we aim for noninferiority in terms of disease activity, as measured by the difference in the mean DAS28 score between the two study groups. Furthermore, we aim for superiority in terms of the number of outpatient clinic visits, with a lower number indicating a better result. Two sample size calculations are performed to determine an appropriate sample size for both hypotheses.

A sample size of 70 patients in each group has 90% power to detect noninferiority using a one-sided two-sample *t*-test. The margin of noninferiority is −0.3, and it corresponds to half of the minimal clinically important difference of 0.6, according to the EULAR response criteria. The true difference between means is assumed to be 0.0. The significance level (alpha) of the test is .05. The data are drawn from populations with a standard deviation of 0.60, which can be generally assumed in a stable group of patients with RA.

As the number of visits will be analyzed and each patient might have multiple visits, the sample size calculation needs to take the exposure-adjusted rate into consideration to power the study. Therefore, a Poisson test has been performed. The expected decrease in visits is approximately 50%, as a recent telemonitoring study in RA showed a decrease in visits of 58% [23]. A sample size of 22 patients in each group has 90% power at the 5% significance level to detect a difference of 25% (half the expected difference) with the use of a one-sided two-sample Poisson test. The exposure time is set at 6 months in the calculation, but with an actual follow-up of 1 year, the power will be even higher.

A sample size of 70 patients in each group will have sufficient power for both the clinical outcome and reduced appointment frequency. Considering these calculations and accounting for follow-up loss of 20% of patients, we plan to include 88 patients in each group (total 176 patients).

### Randomization Procedure

Participants will be randomized 1:1 to self-initiated care with our app (intervention) or standard care (control). Randomization and allocation will be performed by the author BS, using a web-based randomization tool (Castor EDC, Ciwit BV, Amsterdam, the Netherlands) to obtain variable blocks of two, four, or six, with stratification for treatment (conventional or biological DMARD). Castor builds the allocation sequence and performs randomization for the researcher, which can be considered centralized randomization, without the risk of allocation bias [24].

### Blinding

The 28-joint tender and swollen joint count for the primary outcome measure will be determined by a blinded research nurse or rheumatology resident. These individuals have no treatment relationship with the participants and will be instructed not to look at the patient files prior to the examination. The nurse or resident will be called during the study visit to perform the examination. As in most eHealth trials, it will not be possible to blind patients and health care providers caring for their patients to treatment allocation.

### Outcome Measures

The primary outcome measures are health care utilization, as measured by the number of outpatient clinic visits with a rheumatologist during the 12-month trial period, and disease activity, as measured by the DAS28-ESR at 12 months. A list of secondary outcome measures and their assessment time points are presented in Table 2 and Figure 5, respectively. The key secondary outcomes include cost evaluation of the intervention and adherence. Health care costs in the intervention group will be compared with usual care costs from a societal perspective as measured using the Treatment Inventory of Costs in Psychiatric Patients questionnaire, which is adjusted for use in RA patients, and medical information retrieved from the Reade EMR system [25]. Furthermore, explorative analysis will be performed to evaluate the relationship between user adherence and disease activity.

**Table 2:** Secondary measures.

Secondary measure	Scale
Patient satisfaction	10-point Likert scale
Patient empowerment	Effective Consumer Scale 17
Disease activity	Routine Assessment of Patient Index Data 3
Treatment satisfaction	Treatment Satisfaction Questionnaire for Medication 9
Medication adherence	Compliance Questionnaire for Rheumatology 5
Work productivity	Work Productivity and Activity Impairment
Overall cost	Treatment Inventory of Costs^a^
Adherence	Questionnaire completion rates
Qualitative data	N/A^b^

^a^Adjusted for use in rheumatology.

^b^Not applicable.

## Results

Recruitment is currently underway. We started recruitment in May 2019, and it will continue until the goal of 176 participants is reached. Thus far, 78 patients have been randomized and, empirically, experiences with the app have been positive. Data release in the form of a research paper is estimated by the end of 2021.

## Discussion

### Summary and Strengths

This study will be the first randomized trial to test the safety and efficacy of self-initiated care supported by a smartphone app in patients with RA. Although several apps to monitor disease activity exist and some of these apps have been tested, to date, no studies have reported on the use of an app to support patient-initiated care [26-28]. This study has been performed in accordance with the relevant domains of the model for assessment of telemedicine applications, as advised by the European Commission guidelines, and meets the requirements of the checklist on how to report health interventions using mobile phones [29,30].

In a recent systematic review of mobile apps for monitoring disease activity, a lack of high-quality apps was reported [11]. The review suggested that apps should use validated questionnaires and have a user-friendly interface. With the design and redesign strategy and patient feedback in all stages, we were able to develop a mobile app that meets the review requirements. The app is easy for patients to use, visually presents data, and incorporates useful information for physicians. Furthermore, we secured technical support during the trial period, as one of the developers of the app is a part of the project team (FC). Additionally, we have ensured that this system is integrated with the existing Reade EMR system to optimize clinicians’ workflow in their busy daily clinical practice. Integration of the app and its patient-reported outcome (PRO) data with the existing EMR system has enormous research potential. This has been recognized before but is often not accomplished [31].

### Expectations

We anticipate that the app will facilitate better comanagement of the disease by rheumatologists and patients. This will result in early identification of disease flares and will lead to possibly better interventions for patients requiring treatment adaptation. Moreover, we expect a reduced appointment frequency for patients, as unnecessary consultations will be prevented [23]. Furthermore, self-monitoring will improve patient engagement and empower patients to manage their own illness. We expect a fair amount of missing data from the app owing to varying engagements from our patients [32]. Our primary objective is not to use the PRO data in statistical analyses, but to ensure that the data are useful to patients and rheumatologists for monitoring and understanding the disease. Even if patients enter data only once every 4 weeks, they will still have 4-6 times more data about their disease status when compared with the data obtained on visiting the rheumatology outpatient clinic every 4-6 months.

### Limitations

This study has several potential limitations. First, there is a possibility of a ceiling effect of the secondary outcome measures, as all participants will have low disease activity at baseline and a disease duration of at least 2 years and might score well in several of the secondary outcome measures, as is often the case in the Dutch health care system. Second, the addition of a third arm (patient-initiated care without the app) has been discussed to allow evaluation of the effect of the app. However, we opted against the inclusion of a third arm because this would mean that information about disease activity could be lacking for a full year, which is against current EULAR guidelines. To avoid this unethical design, we selected a design similar to that used in the study by de Jong et al, in which the telemedicine system IBDcoach led to a reduction in outpatient clinic visits when compared with usual care [2]. Third, generalizability is limited for three reasons. Firstly, we only include patients who are in remission, and thus, patients with high disease activity are excluded from this intervention. Nevertheless, we think that this intervention is still very relevant, as there are unmet needs to reduce the number of outpatient clinic visits and detect flares early. Secondly, only patients who own a mobile device and therefore are likely receptive to mobile technology are included. However, we anticipate that this selection excludes only a small percentage of individuals, as the proportion of adults with a smartphone is growing and is already at 87% [16]. Thirdly, the MijnReuma Reade App is only accessible to patients at Reade presently, which limits delivery at scale. We have however granted everyone access to the prototype app, which other hospitals can incorporate into their own EMR systems.

### Conclusion

Following a design, test, and redesign approach, we have developed an app that allows patients with RA to monitor their own disease activity. We anticipate that our app will safely lower the need of patients for outpatient clinic visits. If proven safe and effective in an RCT, our aim is to implement our telemonitoring strategy in the Dutch health care system.

## Appendix

Multimedia Appendix 1 The SPIRIT checklist.

## Abbreviations

DAS28: disease activity score 28
DMARD: disease-modifying antirheumatic drug
EMR: electronic medical record
ESR: erythrocyte sedimentation rate
EULAR: European League Against Rheumatism
FQ: flare questionnaire
IBD: inflammatory bowel disease
PRO: patient-reported outcome
RA: rheumatoid arthritis
RAPID3: Routine Assessment of Patient Index Data 3
RCT: randomized controlled trial
