# Supplementary material for: A Smartphone App for Self-Monitoring of Rheumatoid Arthritis Disease Activity to Assist Patient-Initiated Care: Protocol for a Randomized Controlled Trial
Source: JMIR Res Protoc. 2020 Feb 19;9(2):e15105. doi: 10.2196/15105 (PMC7057822; doi:10.2196/15105)
Supplement: Multimedia Appendix 1 [file resprot_v9i2e15105_app1.pdf]

SPIRIT
STANDARD PROTOCOL ITEMS: RECOMMENDATIONS FOR INTERVENTIONAL TRIALS

SPIRIT 2013 Checklist: Recommended items to address in a clinical trial protocol and related documents*

Section/item	Item No	Description	Elaboration
**Administrative information**			
Title	1	Descriptive title identifying the study design, population, interventions, and, if applicable, trial acronym	Title describes study design, population and intervention
Trial registration	2a	Trial identifier and registry name. If not yet registered, name of intended registry	Trial has been registered. Trial ID: NL7715.
	2b	All items from the World Health Organization Trial Registration Data Set	All items are included in the registration
Protocol version	3	Date and version identifier	Final version (accepted by IRB)
Funding	4	Sources and types of financial, material, and other support	None
Roles and responsibilities	5a	Names, affiliations, and roles of protocol contributors	Names and affiliations are provided in the author section and roles are explained in the subsection Authors’ Contributions
	5b	Name and contact information for the trial sponsor	Reade, Dr. Jan van Breemenstraat 2. 0202421800.

	5c	Role of study sponsor and funders, if any, in study design; collection, management, analysis, and interpretation of data; writing of the report; and the decision to submit the report for publication, including whether they will have ultimate authority over any of these activities	This trial is an investigator initiated study funded by AbbVie. AbbVie had no role in the design of this study and will not have any role during its execution, analyses, interpretation of the data, or decision to submit results.
	5d	Composition, roles, and responsibilities of the coordinating centre, steering committee, endpoint adjudication committee, data management team, and other individuals or groups overseeing the trial, if applicable (see Item 21a for data monitoring committee)	Composition and roles are described in methods section. The study is monitored yearly by research monitors.
**Introduction**			
Background and rationale	6a	Description of research question and justification for undertaking the trial, including summary of relevant studies (published and unpublished) examining benefits and harms for each intervention	Described in introduction.
	6b	Explanation for choice of comparators	Described in introduction - Objective
Objectives	7	Specific objectives or hypotheses	Described in introduction - Objective

Trial design	8	Description of trial design including type of trial (eg, parallel group, crossover, factorial, single group), allocation ratio, and framework (eg, superiority, equivalence, noninferiority, exploratory)	Described in introduction - Objective
**Methods: Participants, interventions, and outcomes**			
Study setting	9	Description of study settings (eg, community clinic, academic hospital) and list of countries where data will be collected.	Described in phase 3 – study procedure.
Eligibility criteria	10	Inclusion and exclusion criteria for participants. If applicable, eligibility criteria for study centres and individuals who will perform the interventions (eg, surgeons, psychotherapists)	Described in phase 3 – Eligibility Criteria.
Interventions	11a	Interventions for each group with sufficient detail to allow replication, including how and when they will be administered	Described in phase 3 - Study Procedure.
	11b	Criteria for discontinuing or modifying allocated interventions for a given trial participant (eg, drug dose change in response to harms, participant request, or improving/worsening disease)	Discontinuation will only take place at the participants request.
	11c	Strategies to improve adherence to intervention protocols, and any procedures for monitoring adherence (eg, drug tablet return, laboratory tests)	Participants will be contacted if they have not filled out a questionnaire in 4 weeks. The adherence to the protocol itself is one of the outcomes.

	11d	Relevant concomitant care and interventions that are permitted or prohibited during the trial	Patients may not participate in another trial at Reade as that might influence the number of outpatient clinic visits, this has been accordingly documented in the eligibility criteria. No other restrictions (aside the exclusion criteria) are defined.
Outcomes	12	Primary, secondary, and other outcomes, including the specific measurement variable (eg, systolic blood pressure), analysis metric (eg, change from baseline, final value, time to event), method of aggregation (eg, median, proportion), and time point for each outcome. Explanation of the clinical relevance of chosen efficacy and harm outcomes is strongly recommended.	Outcomes are well defined and logically derived from our hypothesis' as explained in the introduction.
Participant timeline	13	Time schedule of enrolment, interventions (including any run-ins and washouts), assessments, and visits for participants. A schematic diagram is highly recommended (see Figure) [V]	Provided in phase 3 - timeline
Sample size	14	Estimated number of participants needed to achieve study objectives and how it was determined, including clinical and statistical assumptions supporting any sample size calculations	Described in phase 3 - Power analysis
Recruitment	15	Strategies for achieving adequate participant enrolment to reach target sample size.	Described in phase 3 – Study protocol

Methods: Assignment of interventions (for controlled trials)

Allocation:

Sequence generation	16a	Method of generating the allocation sequence (eg, computer-generated random numbers), and list of any factors for stratification. To reduce predictability of a random sequence, details of any planned restriction (eg, blocking) should be provided in a separate document that is unavailable to those who enrol participants or assign interventions	Described in phase 3 - Randomization procedure.
Allocation concealment mechanism	16b	Mechanism of implementing the allocation sequence (eg, central telephone; sequentially numbered, opaque, sealed envelopes), describing any steps to conceal the sequence until interventions are assigned	Described in phase 3 - Randomization procedure.
Implementation	16c	Who will generate the allocation sequence, who will enrol participants, and who will assign participants to interventions	Respectively Castor, [B.S.] and Castor. As described in phase 3 - Randomization procedure.
Blinding (masking)	17a	Who will be blinded after assignment to interventions (eg, trial participants, care providers, outcome assessors, data analysts), and how?	After allocation no blind will be maintained. Described in phase 3 – Blinding.

	17b	If blinded, circumstances under which unblinding is permissible, and procedure for revealing a participant's allocated intervention during the trial [not applicable]	n.a.

Methods: Data collection, management, and analysis

Data collection methods	18a	Plans for assessment and collection of outcome, baseline, and other trial data, including any related processes to promote data quality (eg, duplicate measurements, training of assessors) and a description of study instruments (eg, questionnaires, laboratory tests) along with their reliability and validity, if known. Reference to where data collection forms can be found, if not in the protocol	Described in phase 3 - study procedure and outcome measures (figure 6)
	18b	Plans to promote participant retention and complete follow-up, including list of any outcome data to be collected for participants who discontinue or deviate from intervention protocols	Described in phase 3 - study procedure

Data management	19	Plans for data entry, coding, security, and storage, including any related processes to promote data quality (eg, double data entry; range checks for data values). Reference to where details of data management procedures can be found, if not in the protocol	Data will be recorded on site directly into thea web-based database CASTOR EDC. The database will be used to monitor the progression of the study and the completeness of the data. The principale investigator (WB), research monitors, the inspection for health and youth, and co-investigators have access to the source data, and the key of the code is safeguarded by the PI and a the PhD student [(BS)]. The code will consist of a three-3 letter identification (with letters from the study, SMR) combined with a code that is based on on the basis ofthe inclusion- sequence (SMR#001 and up). This code will be registered for each participant in a separate database. All data will be stored on the servers at the study location (Reade). The study is monitored according to the NFU (Netherlands Federation of University Medical Centers) standards that are applicable in the Netherlands.
Statistical methods	20a	Statistical methods for analysing primary and secondary outcomes. Reference to where other details of the statistical analysis plan can be found, if not in the protocol.	For the two primary outcome measures, the mean number of outpatient clinic visits in the 12 months trial period and the mean DAS28 at T=12 months will be compared between the intervention and the control group.
	20b	Methods for any additional analyses (eg, subgroup and adjusted analyses)	For each outcome measure, we will also adjust for disease duration and activity at baseline, and sex and any variables that are statistically different from each other in unadjusted analyses.
	20c	Definition of analysis population relating to protocol non-adherence (eg, as randomised analysis), and any statistical methods to handle missing data (eg, multiple imputation)	The primary analysis will be using both intent-to-treat (ITT) and per protocol (PP) analysis. In the ITT analysis, missing data (if over 10%) will be imputed using a random intercept mixed model.

Methods: Monitoring

Data monitoring	21a	Composition of data monitoring committee (DMC); summary of its role and reporting structure; statement of whether it is independent from the sponsor and competing interests; and reference to where further details about its charter can be found, if not in the protocol. Alternatively, an explanation of why a DMC is not needed	No DMC is needed since the risk analysis placed the study in the ‘neglectable risk category'
	21b	Description of any interim analyses and stopping guidelines, including who will have access to these interim results and make the final decision to terminate the trial	As the risk of the study is neglectable no stopping guidelines have been predefined. The authors (BS and WB) will have access to the interim results, which will be made public. WB has the final decision to terminate the trial.
Harms	22	Plans for collecting, assessing, reporting, and managing solicited and spontaneously reported adverse events and other unintended effects of trial interventions or trial conduct	Adverse events will be collected during outpatient clinic visits (as per use). No extra adverse events are expected.
Auditing	23	Frequency and procedures for auditing trial conduct, if any, and whether the process will be independent from investigators and the sponsor	No predefined plan for audits has been made. Monitoring will take place at the start, each year during and at the end of the trial.
**Ethics and dissemination**			
Research ethics approval	24	Plans for seeking research ethics committee/institutional review board (REC/IRB) approval	Described in Phase 3 - Study Procedure.

Protocol amendments	25	Plans for communicating important protocol modifications (eg, changes to eligibility criteria, outcomes, analyses) to relevant parties (eg, investigators, REC/IRBs, trial participants, trial registries, journals, regulators)	In case of important protocol modification(s) the ethics committee will be contacted and, in that case, the alterations will be (clearly) described in the manuscript.
Consent or assent	26a	Who will obtain informed consent or assent from potential trial participants or authorised surrogates, and how (see Item 32)	B. Seppen. As described in Phase 3 – study procedure.
	26b	Additional consent provisions for collection and use of participant data and biological specimens in ancillary studies, if applicable	N.a.
Confidentiality	27	How personal information about potential and enrolled participants will be collected, shared, and maintained in order to protect confidentiality before, during, and after the trial	See response in box 19.
Declaration of interests	28	Financial and other competing interests for principal investigators for the overall trial and each study site	Described in Conflicts of Interest section
Access to data	29	Statement of who will have access to the final trial dataset, and disclosure of contractual agreements that limit such access for investigators	See response in box 19.

Ancillary and post-trial care	30	Provisions, if any, for ancillary and post-trial care, and for compensation to those who suffer harm from trial participation	N.a.
Dissemination policy	31a	Plans for investigators and sponsor to communicate trial results to participants, healthcare professionals, the public, and other relevant groups (eg, via publication, reporting in results databases, or other data sharing arrangements), including any publication restrictions	Trial results will be communicated towards the public in the form of a research paper.
	31b	Authorship eligibility guidelines and any intended use of professional writers	Author roles are defined in section Authors' Contributions. No professional writers will be used.
	31c	Plans, if any, for granting public access to the full protocol, participant-level dataset, and statistical code	We plan to grant full access to the protocol and participant level dataset after the relevant material has been published.
**Appendices**			
Informed consent materials	32	Model consent form and other related documentation given to participants and authorised surrogates	A model informed consent form can be requested from the first author. (The informed consent forms are in Dutch).
Biological specimens	33	Plans for collection, laboratory evaluation, and storage of biological specimens for genetic or molecular analysis in the current trial and for future use in ancillary studies, if applicable	N.a.

*It is strongly recommended that this checklist be read in conjunction with the SPIRIT 2013 Explanation & Elaboration for important clarification on the items. Amendments to the protocol should be tracked and dated. The SPIRIT checklist is copyrighted by the SPIRIT Group under the Creative Commons "Attribution-NonCommercial-NoDerivs 3.0 Unported" license.
